# Risk factors for suicide in Bangladesh: case–control psychological autopsy study

**DOI:** 10.1192/bjo.2020.152

**Published:** 2020-12-16

**Authors:** S. M. Yasir Arafat, M. A. Mohit, Mohammad S. I. Mullick, Russell Kabir, Murad M. Khan

**Affiliations:** Department of Psychiatry, Enam Medical College and Hospital, Bangladesh; Department of Psychotherapy, National Institute of Mental Health, Bangladesh; Department of Psychiatry, Bangabandhu Sheikh Mujib Medical University, Bangladesh; School of Allied Health, Faculty of Health, Education, Medicine and Social Care, Anglia Ruskin University, UK; Department of Psychiatry, Aga Khan University, Pakistan

**Keywords:** Risk factors, suicide, Bangladesh, case–control study, psychological autopsy

## Abstract

**Background:**

Suicide is an important, understudied public health problem in Bangladesh, where risk factors for suicide have not been investigated by case–control psychological autopsy study.

**Aims:**

To identify the major risk factors for suicide in Dhaka, Bangladesh.

**Methods:**

We designed a matched case–control psychological autopsy study. We conducted a semi-structured interview with the next-of-kin of 100 individuals who died by suicide and 100 living controls, matched for age, gender and area of residence. The study was conducted from July 2019 to July 2020.

**Results:**

The odds ratios for the risk factors were 15.33 (95% CI, 4.76–49.30) for the presence of a psychiatric disorder, 17.75 (95% CI, 6.48–48.59) for life events, 65.28 (95% CI, 0.75–5644.48) for previous attempts and 12 (95% CI, 1.56–92.29) for sexual abuse.

**Conclusions:**

The presence of a psychiatric disorder, immediate life events, previous suicidal attempts and sexual abuse were found as significant risk factors for suicide in Dhaka, Bangladesh.

Suicide is a leading cause of death worldwide, and identifying risk factors has been considered as the key initiative of suicide prevention.^1^ It has been widely accepted that about 90% of people who die by suicide suffer from at least one psychiatric disorder.^1–3^ Hence, the treatment of psychiatric disorders has been recommended as an important prevention strategy.^1–3^

Bangladesh is a lower middle-income country in South-East Asia, with a high density of population. Dhaka, the capital city, is one of the fastest-growing megacities in the world, and is also the largest city in Bangladesh. It has a population of over 20 million, with a density of about 23 234 people per square kilometer.^4^ Suicide is a neglected public health problem in Bangladesh. There is no national suicide surveillance system, dedicated suicide database or national suicide prevention strategy.^5^ There is a wide variation of suicide rates between different studies and the World Health Organization (WHO) suicide data. As per the WHO report of 2014, the global suicide rate was 7.8 per 100 000 population in both genders, whereas it was 8.7 per 100 000 population in females and 6.8 per 100 000 population in males in 2012 in Bangladesh.^5^ This female predominance has also been revealed by other studies, although the rate varies widely.^5^ Furthermore, there is a strong likelihood of underreporting of suicidal events in the country. All suicide events are supposed to be reported to the police, and the legal authority gives the verdict of suicide.

Risk factors for suicide have not been studied systematically in Bangladesh. The few studies that have been carried out show different sociodemographic and risk factors compared with Western countries.^5^ Female gender, age <30 years and immediate emotionally charged events, rather than mental disorders, have been reported as risk factors in the country.^5,6^ As a result, few and sporadic suicide prevention activities have been initiated, with no central strategy or harmonisation.^7^ With this background, there is an immediate and unmet need to determine the risk factors for suicide in Bangladesh, to formulate a national suicide prevention strategy. Therefore, we aimed to identify the major risk factors for suicide in Dhaka, Bangladesh.

## Method

### Study design

This was a matched case–control psychological autopsy study conducted between July 2019 and July 2020. We conducted a face-to-face semi-structured interview with the next-of-kin of 100 purposively selected individuals who died by suicide and 100 living controls, matched by age, gender and area of residence in Dhaka city.

### Suicide cases

A list of suicide cases with contact details of the deceased (family members, witnesses and police) was obtained from the Department of Forensic Medicine and Toxicology, Dhaka Medical College, and Shaheed Suhrawardy Medical College, Dhaka. The list of cases that were confirmed as suicide after medico-legal autopsy between January 2019 and February 2020 was collected. We contacted the relatives of 358 individuals who died by suicide, to complete the 100 interviews. From the collected list we interviewed the next of kin, and those who agreed to talk with us provided informed written consent. The response rate was 27.93%. The response rate was lower for several reasons: in some cases, family members were not living in Dhaka (*n* = 53, 14.8%); next-of-kin could not always be traced (*n* = 38, 10.6%); some respondents did not want to talk about the matter (98, 27.4%) and in some cases, the individual's address could not be traced (*n* = 69, 19.3%). Stigma, misinformation and fear of legal and social harassment could be reasons for uncooperativeness in the interviews.

### Controls

Age (±2 years), gender and area of residence were matched between cases and controls. The controls were from a close neighbourhood of 25 houses in the same street. The next street was searched if we did not get an appropriate control from the same street. The controls were not present during the interview. We faced similar difficulties in interviewing the next-of-kin of the controls as we faced in the cases. We had to approach 409 prospective controls to complete 100 interviews. The response rate was 24.44%.

### Instruments

We adopted the instrument used in the Karachi Suicide Study^8^ for studying the demographic variables of the cases/controls and informants, past psychiatric history, medical history, psychiatric history of first-degree relatives, details of previous attempts of self-harm, details of the suicide, educational attainment and work conditions, social network and relationship issues, perceived key factors and principal ways of prevention of individual cases, and details of the interview.^8^

Psychiatric illness was investigated with the Structured Clinical Interview for DSM-IV Axis I Disorders (SCID-I).^9^ A detailed treatment history was recorded. The Structured Clinical Interview for DSM-IV Personality Disorders (SCID-II) was used to identify personality disorders.^10^ The SCID-II was used because of a lack of a standard, culturally validated instrument to diagnose personality disorder. Moreover, the interview was conducted by a psychiatrist and SCID-I was used for axis I diagnosis, which ensures the use of instruments from the same classification system of mental disorders. Life events during the past 2 days, month and year were identified.^11,12^ Life events were taken from Paykel's Life Events Schedule.^13^ We also added some significant life events that were not listed in the schedule (e.g. sexual harassment), but are considered significant in Bangladesh. We considered eve-teasing, indecent sexual assault and rape, along with its social consequences, as sexual harassment.

### Interview techniques

Initial contact was made over the mobile phone with the family members of the person who had died by suicide. Once the contact was made, study objectives and processes were discussed in a culturally appropriate manner, and an appointment to visit the house was sought. Then, the visit to the house was performed and a face-to-face semi-structured interview was conducted. A total of nine (four cases, five controls) next-of-kin visited the office of the first author. In several cases, the interview was conducted in front of a local journalist (*n* = 7), local leaders (*n* = 5) and police (*n* = 2). Many times, several family members (*n* = 65) were present. The key informants were parents (43 cases, 49 controls), spouses (24 cases, 31 controls), siblings (10 cases, 4 controls), second-degree relatives (10 cases, 2 controls), children (5 cases, 7 controls), acquaintances (6 cases, 6 controls) and others (betrothed, professionals (2 cases, 1 control)). The primary informant was noted as the responders. However, information was collected, checked and verified from as many respondents as possible. All interviews were conducted by the first author. The timing of the interview ranged from 6 weeks to 6 months after the suicide. A typical interview took 1.5–2 h for the cases and 1–1.5 h for the controls.

### Statistical analysis

For the statistical analysis, we used IBM SPSS version 27 for Windows and Microsoft Excel version 2010. A significance level of *P* < 0.05 was considered statistically significant. Continuous variables were presented as mean ± s.d. Demography and psychiatric diagnoses were presented as frequency and percentages. The dependent variable was the case (suicide) or control status, and independent variables were assumed risk factors. Univariate conditional logistic regression analysis was performed to extract the matched odds ratios and their 95% confidence intervals.

### Ethics statement

The study was conducted in accordance with the ethical standards of the Helsinki Declaration of 1975, as revised in 2008. It was approved by the ethical committee of the National Institute of Mental Health, Dhaka on 20 May 2019 (approval number NIMH/2019/1053). Informed written consent was taken from all respondents.

## Results

### Demographic characteristics

Of the 100 cases, there were 49 males and 51 females. The mean age was 26.30 (±12.36) years for the cases and 26.68 (±11.96) for the controls. The range of age was 9–75 years for the cases and 8–68 years for the controls. Further details are presented in Table 1. There was no significant difference in marital status between the cases and controls. However, there was a difference noted in type of marriage. Choice marriage was more common among suicide deaths (*χ*^2^ = 9.21, *P* = 0.00). No difference was noted regarding religion; however, less religiosity was noted among suicide deaths (*χ*^2^ = 4.88, *P* = 0.03). A total of 64 cases had low socioeconomic status (*n* = 56 controls), 27 had a middle socioeconomic class (*n* = 39 controls) and 9 had an upper socioeconomic class (*n* = 5 controls). Socioeconomic status was assessed based on the monthly income and condition of the residence. A total of 72 cases were living in the peripheral part of Dhaka city and 28 cases were living in the central part of the city.

**Table 1:** Demography of cases and controls

	Cases	Controls	*χ*^2^, *P*-value
Age group, years			
8–20	41	37	*χ*^2^ = 0.82
21–30	33	36	*P* = 0.91
31–40	14	17	
≥40	12	10	
Marital status			
Married	44	41	*χ*^2^ = 0.184
Others	56	59	*P* = 0.67
Type of marriage			
Arranged and both	17	29	*χ*^2^ = 9.21
Choice	23	9	*P* = 0.00
Religious practice			
Highly and moderately religious	9	20	*χ*^2^ = 4.880
Minimally religious	91	80	*P* = 0.03
Children			
No	59	59	*χ*^2^ = 0.059
Yes	23	25	*P* = 0.81
Employment status			
Unemployed	16	5	*χ*^2^ = 6.44
Employed and others	84	95	*P* = 0.01
Educational background			
No education and primary	33	21	
Secondary	55	53	*χ*^2^ = 7.862
Higher and other	12	*26*	*P* = 0.02
Social class			
Upper	9	5	*χ*^2^ = 3.85
Middle	27	39	*P* = 0.14
Lower	64	56	
Total	100	100	

### Circumstances of death

Hanging was the most common method, noted in 70 cases, followed by poisoning (*n* = 15), jumping from high places (*n* = 6), cutting and piercing (*n* = 3), burning (*n* = 3), jumping in front of a moving object (*n* = 2) and firearms (*n* = 1). The majority of the deaths happened at home (*n* = 81), followed by a public place (*n* = 5), hospital (*n* = 4), workplace (*n* = 3), friend's home (*n* = 1) and other (*n* = 6), including a girlfriend's home, in-law's home and orchard. Among the cases, 19 individuals left a suicide note and 56 individuals took active precautions to prevent discovery of the attempt. On query to the respondents regarding why the suicide happened, several reasons were given, including an extramarital affair of the spouse (*n* = 12), premarital affair-related issues (*n* = 12), sexual harassment (*n* = 9), discord with family members (*n* = 10), use of addictive substances (*n* = 7), financial loss and/or insolvency (*n* = 10), spousal discord (*n* = 6), depression (*n* = 8), refusal of a love relationship (*n* = 6), unexpected life events (*n* = 2), forced marriage (*n* = 2), academic failure (*n* = 3), unemployment (*n* = 3), psychiatric illness (*n* = 2) and physical illness (*n* = 2). Several factors arose regarding how an index suicide could be prevented, including legal action and protection (*n* = 9), help from a mental health professional (*n* = 13), good child-rearing practice (*n* = 3), stable income (*n* = 9), financial solvency to provide a dowry (*n* = 2), loyal and good spousal relationship (*n* = 5), academic success (*n* = 2), accepting the love relationship (*n* = 9), avoiding child marriage (*n* = 1), adequate care during a time of crisis (*n* = 10), personal coping (*n* = 3) and good friendship (*n* = 1).

### Psychiatric illness

A total of 61% of cases had any form of psychiatric disorder, compared with 13 controls. About 57% of cases had at least one axis I disorder and 14% had a personality disorder. Among the axis I disorders, 44% had major depressive disorder (MDD), 9% had substance misuse (amphetamine use disorder), 3% had adjustment disorder, 4% had acute stress disorder and 1% had schizophrenia. In comparison, 11% of controls had an axis I disorder and 2% had a personality disorder. Two cases had an axis I disorder with comorbidity of substance misuse and personality disorder. Five cases had comorbid axis I disorder and substance misuse, seven had comorbid axis I disorder and personality disorder and five had comorbid substance misuse and personality disorder. Among the cases, five persons with MDD, seven with amphetamine use disorder and one with schizophrenia were previously diagnosed. The remainder were diagnosed during the interview. The previously diagnosed cases were consulting psychiatrists irregularly and were not treatment-adherent, except for one individual with MDD, who was visiting a psychiatrist regularly and taking medications as per the psychiatrist's suggestion.

### Life events

For cases, 91% experienced life events, and for controls, only 24% experienced the same. Life events were present in 89 cases and 15 controls during the past 48 hours, 50 cases and 16 controls during the past month, and 26 cases and 13 controls during the past year. The life events for the cases were increased arguments with a resident family member (*n* = 10), academic failure (important examination or course) (*n* = 9), broken engagement (*n* = 9), increased arguments with spouse (*n* = 8), sexual harassment (*n* = 8), starting an extramarital affair (*n* = 7), taking a large loan (more than half of a year's earnings) (*n* = 6), spousal extramarital affair (*n* = 5), death of a spouse (*n* = 3), multiple events (*n* = 3), business failure (*n* = 2), fired (*n* = 2), marital separation owing to an argument (*n* = 2), major personal physical illness (hospital admission or 1 month off work) (*n* = 2), moderate financial difficulties (bothersome but not serious, i.e. increased expenses, trouble from bill collectors) (*n* = 2), divorce (*n* = 1), lawsuit (*n* = 1), child married against respondent's wishes (*n* = 1), increased arguments with betrothed or partner (*n* = 1), an argument with a non-resident family member (in-laws, relatives) (*n* = 1), separation from a significant person (close friend or relative) (*n* = 1), moving to another city (*n* = 1), marriage (*n* = 1) and moving within the same city (*n* = 1). Relationship problems with spouses was more common among the cases compared with the controls, and this was statistically significant (*χ*^2^ = 13.041, *P* = 0.00).

### Risk factors

The univariate conditional logistic regression analysis results are presented in Table 2. According to the results, the presence of an immediate life event, psychiatric disorder, previous attempt, sexual abuse, unemployment and physical abuse were reported as significant risk factors (Table 2).

**Table 2:** Risk factors

Risk factor	Cases	Controls	Odds ratio, 95% CI	*P*-value
Past suicide attempt				
No and others	86	99	1	
Yes	14	1	65.28 (0.75–5644.48)	0.066
Physical disability				
Absent and others	96	99	1	
Present	4	1	4.00 (0.45–35.79)	0.215
Physical abuse				
No and others	87	95	1	
Yes	13	5	3.00 (0.96–9.30)	0.057
Sexual abuse				
No and others	88	99	1	
Yes	12	1	12.00 (1.56–92.29)	0.017
Life events				
Absent	9	76	1	
Present	91	24	17.75 (6.48–48.59)	<0.001
Psychiatric disorder				
Absent	39	87	1	
Present	61	13	15.33 (4.76–49.30)	<0.001
Axis I disorder				
Absent	43	91	1	
Present	57	11	16.33 (5.09–52.40)	<0.001
Major depressive disorder				
Absent	56	94	1	
Present	44	6	39.00 (5.36–283.86)	<0.001
Substance misuse				
Absent	91	99	1	
Present	9	1	9.00 (1.14–71.04)	0.037
Personality disorder				
Absent	86	98	1	
Present	14	2	13.00 (1.70–99.37)	0.013
Employment status				
Employed and others	84	95	1	
Unemployed	16	5	4.67 (1.34–16.24)	0.015
Social networks				
Isolated	21	15	2.13 (0.97–4.71)	0.060
Few friends (1–3)	51	40	1.89 (1.04–3.42)	0.036
Many friends (4 or more)	28	45	1	

## Discussion

Risk factors for suicide have been poorly researched in Bangladesh. We aimed to identify the risk factors for suicide, using a matched-pair case–control psychological autopsy design in Dhaka, Bangladesh.

Our findings suggest that suicides have been happening in the early adulthood of life, as the mean age was 26.30 (±12.36) years. However, suicides in extreme ages have also been noticed, i.e. 9–75 years. A recent systematic review also reported a similar finding regarding age distribution.^5^ Studies also found that married females living in low socioeconomic status in the country are dying by suicide more frequently, although the difference was not prominent in the current study.^5,6^ The total population structure is also comparable with the study sample, as the most recent census revealed a nearly equal number of men and women, and >60% of the population were aged <30 years. However, our findings revealed that marriage by choice was more common among the suicide cases, which was statistically significant. Further studies are needed to explain the finding. We speculate that the observation may be because of emotional instability during personal life events. Moreover, many instances of extramarital affairs were not accepted by the parental family members, resulting in poor social capital. Therefore, in response to any devastating personal events, the individual may feel alone and think of ending their life. An un-congenial attitude to the premarital love relationship could lead to fatal acts.

Hanging was the most common method of suicide (*n* = 70), followed by poisoning (*n* = 15), which is in accordance with previous studies.^5^ However, the findings may be different in rural areas, where poisoning may be more common than in the present study.

A total of 61% of cases had any form of psychiatric disorder, 57% had at least one axis I disorder (MDD 44%, substance misuse 9%) and 14% had a personality disorder. This finding is widely varied from the findings in Western countries, where >90% of suicide victims had psychiatric morbidity.^1–3^ This was found to be 96% in Pakistan,^8^ 88% in India^14^ and 80% in Indonesia.^15^ However, a lower prevalence was reported in several studies in China: Phillips et al reported 63%,^12^ Phillips et al reported 65.5%,^16^ Li et al reported 64%,^17^ Kong and Zhang reported 47% in rural China,^18^ Tong and Phillips reported 62.9%,^19^ Zhang and Zhou reported 75.3%^20^ and Zhang et al reported 48% in rural China.^21^ A recent study in Hungary, by Almasi et al, reported a prevalence of 69.1%.^22^ Other studies from India reported lower prevalence: Gururaj et al reported 42.75% in Bangalore,^23^ and Manoranjitham et al reported 37%.^24^ However, there are strong chances of underreporting of symptoms because of stigma, poor mental health literacy and awareness. Some people could never identify the symptoms of mental disorders in Bangladesh, whereas some others do not want to disclose the psychiatric illness. Depression (MDD) was the most common psychiatric disorder (44%), and similar high proportions for depression were reported in almost every study. Our study revealed nine cases with amphetamine (yaba) use disorder, and none of them were diagnosed with alcohol use disorder, which is also different from Western countries and India, but similar to Pakistan. This finding can be explained by the religious and social structure of Bangladesh, where alcohol is prohibited. There were only 13 cases that were previously diagnosed, and mental health services were used in an irregular fashion, which signifies a large psychiatric treatment gap in the country.

In the current study, 91% of cases experienced life events, and a good proportion of events were related to social factors such as increased arguments with a resident family member, breaking off an engagement, increased arguments with a spouse, sexual harassment, beginning an extramarital affair, a spouse having an extramarital affair, marital separation owing to an argument, child married against respondent's wishes, increased arguments with betrothed or partner, and arguments with a non-resident family member (in-laws). Extrapolation of opinions of respondents regarding the deciding factor of suicides revealed that 57% of the events are related to sexual issues such as extramarital affair of a spouse, premarital affair-related issues, sexual harassment, discord with family members, spousal discord, refusal of a love relationship and forceful marriage. A recent systematic review also revealed nearly similar life events of suicide deaths in the country.^5^ Another case–control study also found psychosexual issues, such as problems with a love relationship, problems with relative marital disharmony and familial discord, as risk factors.^6^ This indicates psychosexual events have an important role in the suicidality of people living in Dhaka city, and further studies are warranted to identify the role more specifically. We speculate that enduring cultural attitudes toward sexuality, marriage, low economic freedom of females, poor mental health literacy, lengthy legal process, unethical use of political influence/or power, extreme income inequality, perceived sense of insecurity and social learning could be important attributing factors.

The study identified immediate life events (odds ratio 17.75), psychiatric disorders (odds ratio 15.33), MDD (odds ratio 39), personality disorder (odds ratio 13), amphetamine use disorder (odds ratio 9), sexual abuse (odds ratio 12), physical abuse (odds ratio 3), physical disability (odds ratio 4), past suicidal attempt (odds ratio 65.28) and unemployment (odds ratio 4.67) as significant risk factors (Table 2). Similar risk factors were identified in the other studies; however, sexual abuse and sexual relationship events, such as a premarital love affair, extramarital affair, sexual harassment, rape, eve-teasing and disseminating sexual videos on the internet, have been found in the study differently.

### Implications

The study revealed a lower rate of prevalence of mental disorders among the suicide cases compared with Western countries, and also revealed relationships and psychosexual aspects related to suicide. Preventive strategies should focus on increasing the awareness regarding mental health and suicide, immediate distress coping, an educational approach for the general population (with special attention to immediate distress management), initiation of widely available hotlines, holistic approaches to stop sexual harassment and prioritising training for general practitioners to identify the depression.

### Future direction

Further studies are warranted to explore the psychosexual and emotional relationship influences on suicide for a better understanding and implementation of prevention strategies. The distribution of risk factors in respect to sex could have differences. For females, sexual harassment may be a prominent risk factor before marriage whereas spousal discord could be a deciding factor after marriage. The relationship between age and psychiatric morbidity among suicide cases could another area of interest. A national suicide database, suicide surveillance system, and national suicide prevention program should be initiated. A nationwide psychological autopsy study may depict a different, however complete picture.

### Strengths and limitations

This is the first study that systematically assessed the risk factors of suicide in Bangladesh with a case–control psychological autopsy design, structured instruments and adequate sample size. The controls were matched by age, gender and area of residence. However, prudent interpretation is needed when generalising the study findings, as it was conducted only in the capital city, which has a different distribution from the rural part of the country. The response rate was low in both the case and control groups, and cases were chosen in a non-randomised manner. No structured instrument was used to assess religious practices and social status. There might have a chance of recall biases of the next-of-kin, and masking of case–control status was not done. Moreover, because of stigma and low mental health literacy, psychiatric symptoms and substance misuse history could be hidden.

The presence of a psychiatric disorder, immediate life events, previous suicidal attempts and sexual abuse were all found to be significant risk factors of suicide. Relationship events were important precipitating events, and issues related to emotional and sexual relationships had significant dominance as life events. Measures are warranted to change the social understanding of sex, sexuality and sexual harassment so that these life events could be crossed.

## Data Availability

The data that support the findings of this study are available on request from the corresponding author, S.M.Y.A. The data are not publicly available because they containing information that could compromise the privacy of research participants.
